# Antigenotoxic properties of the halophyte *Polygonum maritimum* L. highlight its potential to mitigate oxidative stress-related damage

**DOI:** 10.1038/s41598-022-20402-5

**Published:** 2023-03-06

**Authors:** Daniela Oliveira, Maria Inês Dias, Lillian Barros, Luísa Custódio, Rui Oliveira

**Affiliations:** 1 Centre for the Research and Technology of Agro-Environmental and Biological Sciences (CITAB), Department of Biology, University of Minho, Campus de Gualtar, 4710-057 Braga, Portugal; 2 Centre of Molecular and Environmental Biology (CBMA), Department of Biology, University of Minho, Campus de Gualtar, 4710-057 Braga, Portugal; 3 Centro de Investigação de Montanha (CIMO), Instituto Politécnico de Bragança, Campus de Santa Apolónia, 5300-253 Bragança, Portugal; 4 Centre of Marine Sciences (CCMAR), University of Algarve, Campus de Gambelas, 8005-139 Faro, Portugal

**Keywords:** Chemical biology, Plant sciences

## Abstract

Long-term exposure to dietary xenobiotics can induce oxidative stress in the gastrointestinal tract, possibly causing DNA damage and contributing to the initiation of carcinogenesis. Halophytes are exposed to constant abiotic stresses, which are believed to promote the accumulation of antioxidant metabolites like polyphenols. The aim of this study was to evaluate the antioxidant and antigenotoxic properties of the ethanol extract of the aerial part of the halophyte *Polygonum maritimum* L. (PME), which can represent a dietary source of bioactive compounds with potential to attenuate oxidative stress-related damage. The PME exhibited a high antioxidant potential, revealed by the in vitro capacity to scavenge the free radical DPPH (IC_50_ = 2.29 ± 0.10 μg/mL) and the improved viability of the yeast *Saccharomyces cerevisiae* under oxidative stress (*p* < 0.001, 10 min). An antigenotoxic effect of PME against H_2_O_2_-induced oxidative stress was found in *S. cerevisiae* (*p* < 0.05) with the dominant deletion assay. In vitro colorimetric assays and LC-DAD-ESI/MS^n^ analysis showed that PME is a polyphenol-rich extract composed of catechin, (epi)catechin dimer and trimers, quercetin and myricetin glycosides. Hence, *P. maritimum* is a source of antioxidant and antigenotoxic metabolites for application in industries that develop products to provide health benefits.

## Introduction

Industrial advances have led to an increasing consumption of foods contaminated with xenobiotics, such as those formed due to food processing and cooking at high temperatures or that arise from direct contact between foods and their packaging, which can perturb homeostasis and contribute to the development of human diseases^1^. Recurrent ingestion of xenobiotics may induce inflammation in the gastrointestinal tract, leading to the production of reactive oxygen species [ROS, e.g., hydrogen peroxide (H_2_O_2_) and hydroxyl radical (^•^OH)] and reactive nitrogen species (e.g., nitric oxide)^2^. In addition, inflammatory cytokines are produced, and these can activate enzymes capable of generating oxidants^3^. The redox imbalance caused by this adverse situation may lead to oxidative stress and consequent oxidative damage of cellular constituents, such as proteins, lipids and DNA, potentially promoting the development of oxidative stress-related illnesses. These include gastrointestinal diseases and cancers^2^, neurodegenerative ailments^3^, diabetes^4^ and others. Some xenobiotics can be carcinogenic due to their capability to induce DNA damage directly or after metabolic activation by generating carcinogenic metabolites able to interact with DNA and ROS that can cause oxidative damage^5^. If not repaired, DNA damage can induce mutations and genomic instability and may eventually lead to cancer risks.

Polyphenol rich-diets have been linked to health benefits that include lower mortality risk and preventive effects against diseases such as cancers of the gastrointestinal tract^6^. These benefits are often attributed to the antioxidant and anti-inflammatory properties of polyphenols^7^. Additionally, these phytochemicals have been associated with protection against DNA damage^8^. Halophytes are unique plant species able to live in saline environments due to the development of adaptive responses that allow them to counteract the extreme adversity inherent to their habitat, such as the accumulation of non-enzymatic antioxidants to neutralize ROS and prevent oxidative damage^9^. These antioxidants include vitamins, carotenoids and polyphenols, which are responsible for beneficial nutritional and medicinal properties of such plants. In fact, local communities in the coastal areas, such as those in the Mediterranean basin, have been using halophytes as food or in traditional medicine to relieve several human ailments, including cancer and diabetes^10^. Due to the harsh conditions that halophytes have to endure to survive, it is not surprising that these plants contain a higher level of phenolic compounds than glycophytes which correlates to a higher antioxidant activity that, in some cases, is even more potent than that of synthetic antioxidants^11^.

*Polygonum maritimum* L., or sea knotgrass, is a halophyte that can be found on the sandy coasts of America, Europe, South Africa and in the Mediterranean region^12^. Previous studies with extracts of *P. maritimum* from the Mediterranean region revealed a high content in phenolic compounds, particularly in flavonoids, which were suggested to contribute to a considerable antioxidant activity^13–15^. Other interesting bioactivities attributed to *P. maritimum* extracts include anti-inflammatory and anti-diabetic^16^, neuroprotective^14^ and anti-microbial^13^, suggesting its potential application in several industrial fields. Although extracts of *P. maritimum* have been previously reported to be rich in phenolic compounds and to display antioxidant properties, to our knowledge there are no reports of its antigenotoxicity. Thus, the aim of this study was to evaluate the antioxidant and antigenotoxic properties of an ethanol extract of *P. maritimum* from the south of Portugal, which could reinforce its potential application in the food and nutraceutical areas.

## Materials and methods

### Plant material and extraction

The aerial part of *Polygonum maritimum* L. was collected in August 2018 at the Faro beach (south of Portugal; coordinates: 37° 0′ 30.0852′′ N, 7° 59′ 45.2616′′ W). The plant species was identified by Dr. Luísa Custódio (Centre of Marine Sciences, University of Algarve, Portugal) and a voucher specimen was kept in the XtremeBio laboratory (voucher code MBH22). All methods used in the experimental research on plants, including the collection of plant material, complied with relevant institutional, national, namely by the Portuguese Institute for Nature Conservation and Forests (ICNF), and international guidelines and legislation. The plant material was washed, oven dried at 40 °C for five days and reduced to a fine powder. The extraction occurred in absolute ethanol in a flask protected from the light at 25 °C and 100 revolutions per minute (rpm) for 24 h. The *P. maritimum* L. aerial part ethanol extract (PME) was filtered (Whatman grade 4 paper and with syringe filter 0.2 µm), the major part of the solvent was evaporated in a Rotavapor under low pressure at 40 °C and 50 rpm, and the remaining solvent in the concentrated extract was evaporated with gaseous nitrogen. The dried extract was stored at  − 20 °C until use.

### Chemical composition

#### Total phenolic content

The total phenolic content (TPC) of PME was estimated by the Folin-Ciocalteu method^17^ adapted for microplate assay. For this assay, 10 μL of sample (25–175 μg/mL; dissolved in absolute ethanol) were mixed with 50 μL of a diluted solution of Folin-Ciocalteu reagent (1:10 *v/v* in deionized water) and 40 μL of Na_2_CO_3_ (75 mg/mL in deionized water), incubated at room temperature in the dark for 1 h, and the absorbance was measured at 760 nm. The assay included blanks and controls, where the Folin-Ciocalteu reagent and Na_2_CO_3_ were replaced by deionized water and the samples by absolute ethanol, respectively. The absorbance values calculated (absorbance = absorbance sample − absorbance blank − absorbance control) were correlated with a gallic acid standard curve to express the TPC of PME in mg of gallic acid equivalents (GAE)/g of dry weight (DW).

#### *Ortho*-diphenol content

The *ortho*-diphenol content (ODC) of PME was determined according to Domínguez-Perles et al.^18^, with some alterations for use in microplate. In brief, 160 μL of sample (150–300 μg/mL; dissolved in 50% ethanol) were added to the microplate, followed by the addition of 40 μL of sodium molybdate (50 mg/mL in 50% ethanol). The microplate was incubated at room temperature in the dark for 15 min and the absorbance was read at 370 nm. Blanks and controls were included in the experiment, where sodium molybdate and samples were substituted by ethanol 50%, respectively. The ODC of PME was expressed in mg of GAE/g of DW by correlating the absorbance values of the samples (absorbance = absorbance sample − absorbance blank − absorbance control) with a gallic acid standard curve.

#### Total flavonoid content

The total flavonoid content (TFC) of PME was estimated using the methodology described by Kumazawa et al.^19^, with minor modifications to adapt for microplate assay. Briefly, 50 μL of AlCl_3_ (20 mg/mL in absolute ethanol) were added to 50 μL of sample (1000–2000 μg/mL; dissolved in absolute ethanol), the mixture was incubated at room temperature in the dark for 1 h and the absorbance was measured at 420 nm. The assay included blanks and controls, where AlCl_3_ and the samples were replaced by absolute ethanol, respectively. The absorbance values (absorbance = absorbance sample − absorbance blank − absorbance control) were correlated with a quercetin standard curve to express the TFC of PME in mg of quercetin equivalents (QE)/g of DW.

#### Liquid chromatography with diode array detection and electrospray ionization tandem mass spectrometry (LC-DAD-ESI/MS^n^)

##### Standards and reagents

Acetonitrile (99.9%) was of High-Performance Liquid Chromatography (HPLC) grade from Fisher Scientific (Lisbon, Portugal). Formic acid was purchased from Panreac Química S.L.U. (Barcelona, Spain), and phenolic standards were from Extrasynthèse (Genay, France). Water was treated in a Milli-Q water purification system (TGI Pure Water Systems, USA).

##### Phenolic compounds analysis

The phenolic profile of PME was determined in the dried extract re-dissolved in a methanol:water (80:20, *v/v*) mixture by liquid chromatography with diode array detection and electrospray ionization tandem mass spectrometry (LC-DAD-ESI/MS^n^) (Dionex Ultimate 3000 UPLC, Thermo Scientific, San Jose, CA, USA), as previously described by Bessada et al.^20^. For the double online detection, 280 and 370 nm were used as preferred wavelengths for diode array detection (DAD) and in a mass spectrometer (MS) connected to HPLC system via the DAD cell outlet. The MS detection was performed in negative mode, using a Linear Ion Trap LTQ XL mass spectrometer (ThermoFinnigan, San Jose, CA, USA) equipped with an electrospray ionization (ESI) source. The identification of the phenolic compounds was performed using standard compounds, when available, by comparing their retention times, UV–Vis and mass spectra; and also, comparing the obtained information with available data reported in the literature giving a tentative identification. For quantitative analysis, a 7-level calibration curve for each available phenolic standard was constructed based on the UV signal [catechin (*y* = 84.950*x* − 23.200, *R*^2^ = 0.999, LOD (Limit of detection) = 0.17 μg/mL; LOQ (Limit of quantification) = 0.68 μg/mL, peaks 1, 2, 3, 4, and 5), myricetin (*y* = 23287*x* − 581,708, *R*^2^ = 0.9988, LOD = 61.21 µg/mL and LOQ = 185.49 µg/mL, peaks 6 and 7) and quercetin-3-*O*-glucoside (*y* = 34843*x* − 160,173, *R*^2^ = 0.9998; LOD = 0.21 μg/mL; LOQ = 0.71 μg/mL, peak 8)]. For the identified phenolic compounds for which a commercial standard was not available, the quantification was performed through the calibration curve of the most similar available standard. The results were expressed as mg/g of extract DW.

### Eukaryotic model and growth conditions

The budding yeast *Saccharomyces cerevisiae* was selected as eukaryotic cell model for this work. The strains BY4741 (*MATa his3Δ1 leu2Δ0 met15Δ0 ura3Δ0*) and CEN.PK dDEL (*HIS3 Δ::dDEL*; created by Silva et al.^21^) were used for measurement of antioxidant activity in viability assays (colony-forming units, CFU’s) and antigenotoxicity using the dominant deletion (dDEL) assay, respectively. The strain CEN.PK dDEL was created by replacing the *HIS3* gene from the plasmid pPS1 with the dDEL cassette, which is limited by two partial alleles of the *hphMX6Δ* marker that comprise between them sequences with homology and a marker for geneticin (G418) resistance, in the laboratory strain CEN.PK 102-3A (*Mata ura3-52 HIS3 leu2-3112 TRP1 MAL2-8c SUC2*)^21^. Upon double-strand break in the region comprised between the partial alleles of the *hphMX6Δ* marker, the homologous recombination repair pathway is activated, leading to the reversion of the marker. Due to this process, the strain loses the marker for geneticin resistance and becomes resistant to hygromycin B (HygB). The strains BY4741 and CEN.PK dDEL were cultured every week on solid rich medium [YPDA; composed of 1% (*w/v*) yeast extract (Acros Organics), 2% (*w/v*) peptone (BD Bacto), 2% (*w/v*) glucose, 2% (*w/v*) agar (Liofilchem)] and YPDA supplemented with 300 µg/mL geneticin, respectively, and stored at 4 °C. For each experiment, one colony from the cultures at 4 °C was suspended in liquid rich medium (YPD; same composition as YPDA, except agar) and YPD supplemented with 400 µg/mL geneticin for BY4741 and CEN.PK dDEL strains, respectively, and incubated at 30 °C and 200 rpm. Cell proliferation was monitored by measuring the optical density at 600 nm (OD_600_). In the dDEL assay, the recombinant cells were selected on YPDA medium supplemented with 100 µg/mL HygB.

### Antioxidant activity

#### DPPH radical scavenging assay

The capacity of PME to scavenge the free radical 2,2-diphenyl-1-picrylhydrazyl (DPPH) was assessed with an adapted procedure from Mitra and Uddin^22^. In brief, 50 μL of sample (1–10 μg/mL; dissolved in absolute ethanol) were mixed with 100 μL of DPPH solution (0.04 mg/mL), incubated at room temperature in the dark for 20 min and the absorbance was measured at 517 nm. The assay included blanks and controls, where DPPH and samples were replaced by absolute ethanol, respectively. Gallic acid was used as standard. The absorbance values obtained were used to calculate the percentage of DPPH inhibition as follows: (control absorbance − (sample absorbance − blank absorbance))/control absorbance. The IC_50_ values were calculated and represent the concentration required to inhibit 50% of the initial amount of DPPH radical.

#### Cell viability under H_2_O_2_-induced oxidative stress

A liquid culture of BY4741 grown overnight was diluted to OD_600_ = 0.2 and incubated for at least two generations further (~ 4 h; OD_600_ = 0.8). The culture was then divided into aliquots and 100 μL were taken at time 0 min. Next, the aliquots were treated as follows: negative control, 1.3% absolute ethanol; positive control, 5 mM H_2_O_2_ and 1.3% absolute ethanol; control of extract, 250 μg/mL PME; co-incubation, 250 μg/mL PME and 5 mM H_2_O_2_; followed by incubation at 30 °C and 200 rpm. Aliquots of 100 μL were taken at 10, 20 and 30 min after the addition of treatments, serially diluted in sterile deionized water to 10^−4^ and spread on YPDA, followed by incubation at 30 °C for 48 h. The viability (%) was assessed by counting CFU’s and dividing the number of colonies in each time point by the number of colonies at 0 min (before the addition of treatment). Time 0 min was assumed as 100% viability.

### Antigenotoxicity

The antigenotoxicity of PME was evaluated using the dDEL assay according to Silva et al.^21^. Briefly, a liquid culture of CEN.PK dDEL grown overnight was diluted to OD_600_ = 0.2 and incubated for 4 h until OD_600_ = 0.8. The culture was then divided into aliquots, 200 μL were taken at time 0 min, followed by the addition of treatments as described in the previous section, except for the use of 4 mM H_2_O_2_ instead of 5 mM. Then, 200 μL aliquots were taken from each situation after 10 min and diluted to the final volume of 1 mL. The samples were centrifuged at maximum speed for 30 s, 950 μL of supernatant were discarded and the pellet was suspended in 950 μL of YPD, followed by incubation at 30 °C, 200 rpm, for 2 h to guarantee sufficient expression of the *hphMX6* marker. Afterwards, each sample was serially diluted in sterile deionized water to 10^−4^. For each situation, 100 μL from the undiluted samples were placed on YPDA supplemented with 100 μg/mL HygB and 100 μL from the 10^−4^ dilution on YPDA medium, followed by incubation at 30 °C for 48 h. The dDEL recombination frequency was calculated by dividing the number of recombinant colonies on YPDA + HygB medium by the number of viable colonies on YPDA medium, and then normalized to the positive control (4 mM H_2_O_2_).

### Statistical analysis

All data are presented as the mean of three independent experiments with associated standard error of the mean (mean ± SEM). The statistical analysis was performed with the GraphPad Prism (version 8.2.1), using the Student’s t-test to analyze the differences between samples. Differences were considered statistically significant when *p* < 0.05.

## Results and discussion

### Chemical composition

Phenolic compounds are often associated with a strong antioxidant capacity and with other bioactivities of interest, such as anti-inflammatory and antigenotoxic. Thus, the chemical composition of PME was assessed in terms of total phenol, *ortho*-diphenol and flavonoid contents by colorimetric methods. The results indicated that PME has a high content in total phenols and *ortho*-diphenols, and that part of them may be flavonoids (Table 1). The PME showed TPC similar to an ethanol extract of *P. maritimum* from the south of Portugal (241 ± 4 mg GAE/g DW)^15^, but lower than a methanol extract from the Algerian coast (352.49 ± 18.03 mg GAE/g DW)^13^ and higher than methanol extracts of stems (75.9 mg GAE/g DW) and leaves (70.40 mg GAE/g DW) of the same species collected in southern Tunisia^23^, suggesting that the geographical location may have influenced the TPC of *P. maritimum* extracts. Phenolic compounds with *ortho*-diphenol structure are likely to provide a strong antioxidant activity. Hydroxyl groups can neutralize free radicals and their adjacent position in the aromatic ring allows the stabilization of the resultant phenoxyl radical by forming an intramolecular hydrogen bond^24^. Flavonoids are expected to play a major part in the antioxidant capacity of PME, as they are considered main secondary metabolites in the *Polygonum* genus^25^. Accordingly, flavonoids were indicated as the predominant phenolic constituent of a *P. maritimum* aerial part acetone extract from the south of Portugal that revealed antioxidant properties^15^. Flavonoids were detected in PME; however, its TFC value was lower than that of *P. maritimum* methanol extracts from southern Tunisia^23^, perhaps due to differences in the geographical location or harvesting conditions.

**Table 1:** Chemical composition of *P. maritimum* aerial part ethanol extract (PME) determined by in vitro colorimetric assays: total phenolic content (TPC) and *ortho*-diphenol content (ODC) expressed as mg of gallic acid equivalents (GAE)/g of dry weight (DW) and total flavonoid content (TFC) expressed as mg of quercetin equivalents (QE)/g of DW. Data are presented as mean of three independent experiments performed in triplicate with associated standard error of the mean (mean ± SEM).

Sample	TPC (mg GAE/g DW)	ODC (mg GAE/g DW)	TFC (mg QE/g DW)
PME	249.03 ± 1.28	315.71 ± 4.61	17.18 ± 0.56

**Figure 1:**
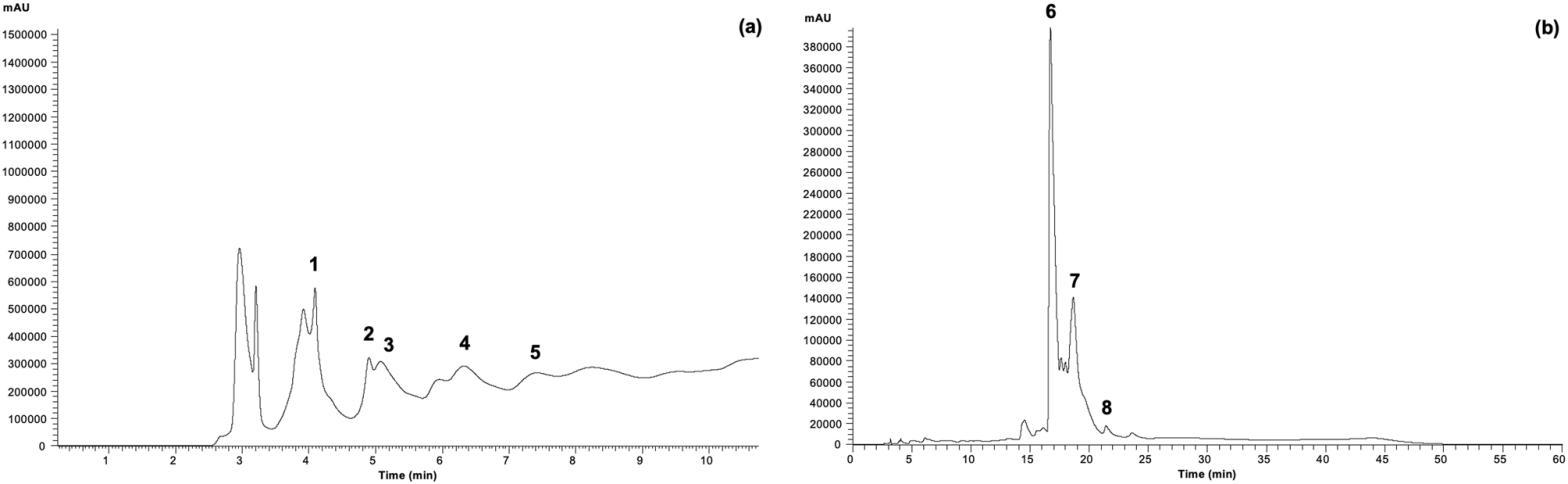
Representative phenolic profile of the *P. maritimum* aerial part ethanol extract recorded at 280 nm (**a**) and 370 nm (**b**). Peak identification is presented in Table 2.

The chemical composition of PME was also analysed by LC-DAD-ESI/MS^n^, which confirmed the presence of phenolic compounds and flavonoids detected in the in vitro colorimetric assays. Eight phenolic compounds were tentatively identified and quantified in the extract (Table 2), revealing the dominance of flavan-3-ols over flavonol glycosides. A representative chromatogram of the phenolic profile of PME, recorded at 280 and 370 nm, is presented in Fig. 1. Flavan-3-ols and flavonol glycosides have been previously described in extracts of *P. maritimum*^12,14,15,23^ and were suggested to be correlated with their biological properties, namely antioxidant, anti-inflammatory, anti-melanogenic and neuroprotective activities. Additionally, β-type (epi)catechin oligomers were found in the rhizome extract of *Polygonum paleaceum* Wall. ex Hook. f.^28^. The concentration of flavan-3-ols was 29.5 ± 1.2 mg/g extract DW, which represented approximately ~ 68% of the total phenolic compounds found (43.3 ± 0.3 mg/g extract DW) and double of the obtained for the flavonols (13.7 ± 0.2 mg/g extract DW, ~ 32% of the total phenolic). Flavan-3-ols detected included catechin (monomer) and four β-type (epi)catechin oligomers (dimer and trimers). Flavonols included two myricetin glycosides and one quercetin glycoside*.* Flavan-3-ols and flavonols have attracted considerable attention due to their recognized bioactivities that include antioxidant, anti-inflammatory, cardioprotective, anticancer, anti-viral and anti-microbial properties^7,29–31^. Overall, these results indicate that PME is composed of phenolic compounds that are likely to provide many valuable properties, including a remarkable antioxidant activity.

**Table 2:** Chemical composition of the *P. maritimum* aerial part ethanol extract determined by liquid chromatography with diode array detection and electrospray ionization tandem mass spectrometry (LC-DAD-ESI/MS^n^). The retention times (Rt), wavelengths of maximum absorption (λ_max_) in the UV–Vis region, main [M–H]^−^ and respective fragment ions (MS^2^), tentative identification, chemical class, and quantification (mg/g of extract DW) of phenolic compounds are presented.

No	Rt (min)	λ_max_ (nm)	[M-H]^-^ (*m/z*)	MS^2^ (*m/z*)	Tentative identification	Chemical class	Quantification (mg/g DW)
1	4.90	278	577	577(72), 575(39), 425(16), 407(10), 289(6), 287(12)	β-type (epi)catechin dimer^26^	Favan-3-ol	3.6 ± 0.2
2	5.08	280	865	739(83), 695(100), 577(74), 575(47), 425(12), 289(10), 287(8)	β-type (epi)catechin trimer^26^	Favan-3-ol	4.4 ± 0.4
3	5.95	280	865	739(74),695(100),577(36), 575(62),425(15),289(12),287(14)	β-type (epi)catechin trimer^26^	Favan-3-ol	4.6 ± 0.1
4	6.32	281/311	289	245(35), 203(15), 137(29)	( +)-Catechin^15,26^	Favan-3-ol	7.5 ± 0.2
5	7.42	280	865	739(74), 695(100), 577(36), 575(62), 425(15), 289(12), 287(14)	β-type (epi)catechin trimer^26^	Favan-3-ol	9.4 ± 0.1
6	16.73	349	463	317(100)	Myricetin-*O*-deoxyhexoside^27^	Flavonol	8.0 ± 0.1
7	18.65	348	463	317(100)	Myricetin-*O*-deoxyhexoside^27^	Flavonol	5.1 ± 0.1
8	21.43	342	447	301(100)	Quercetin-*O*-deoxyhexoside[27]	Flavonol	0.64 ± 0.01
						Total phenolic compounds	43.3 ± 0.3
						Total flavan-3-ols	29.5 ± 1.2
						Total flavonols	13.7 ± 0.2

### Antioxidant activity

The in vitro antioxidant activity of PME was evaluated by measuring its capacity to scavenge the free radical DPPH. The extract showed the capacity to scavenge 50% of the free radical at low concentration (IC_50_ = 2.29 ± 0.10 µg/mL, data not shown), revealing a strong antioxidant effect. The DPPH scavenging capacity of PME was lower than that of the standard used, gallic acid (IC_50_ = 0.86 ± 0.03 µg/mL, *p* < 0.001, data not shown), but higher than the activities reported for phenolic extracts recognized for their antioxidant properties such as *Ginkgo biloba* L. (IC_50_ = 33.91 ± 1.16 µg/mL) or *Camellia sinensis* L. (IC_50_ = 14.50 ± 1.69 µg/mL)^32^, which are often used as standards in in vitro antioxidant assays. In addition, PME revealed higher DPPH scavenging capacity than *P. maritimum* methanol extracts prepared with aerial parts from Algeria (IC_50_ = 7.71 ± 1.88 µg/mL)^13^ or with leaves from the south of Portugal (IC_50_ = 26.0 ± 0.7 µg/mL)^16^, possibly due to differences in the weather conditions at the time of harvesting. The remarkable antioxidant activity observed in our study seems to be a feature of halophytes, although typically not as strong as the one displayed by PME. Studies have reported relatively low IC_50_ values in the DPPH scavenging assay for extracts of halophytes, such as the species *Tamarix gallica* L. (aerial parts methanol extract, IC_50_ = 14.05 ± 0.66 µg/mL)^33^, *Limonium delicatulum* (methanol extracts of leaves and roots, IC_50_ = 10.58 ± 0.18 µg/mL and IC_50_ = 5.79 ± 0.05 µg/mL, respectively)^34^, and *Arthrocnemum indicum* (Willd.) Moq. (shoots ethanol extract, IC_50_ = 7.17 ± 1.26 μg/mL)^35^, associated to a high content in phenolic compounds. Although fruits and vegetables are the common source of polyphenols in the human diet and whose consumption has been associated with many health benefits, in part due to their antioxidant properties, it should be noted that most of these foods do not exhibit an antioxidant activity comparable to that of halophytes like *P. maritimum*. For instance, from a study with methanol extracts of 8 wild edible leafy vegetables, only the species *Cassia tora* (IC_50_ = 9.898 µg/mL) revealed a high antioxidant activity, but still considerably lower than PME, whereas the rest of the 7 vegetables showed lower antioxidant activity as indicated by the higher IC_50_ values that ranged from 33.82 to 45.68 µg/mL^36^. Moreover, methanol extracts of commonly consumed vegetables like spinach (IC_50_ = 200.4 ± 2.1 µg/mL), carrot (IC_50_ = 97.6 ± 2.1 µg/mL), and radish (IC_50_ = 155.7 ± 0.8 µg/mL)^37^ revealed a considerably higher IC_50_ than PME, evidencing their lower antioxidant capacity. Therefore, halophytes like *P. maritimum* could represent a source of polyphenols with more potent antioxidant properties than those that are provided by conventional foods.

**Figure 2:**
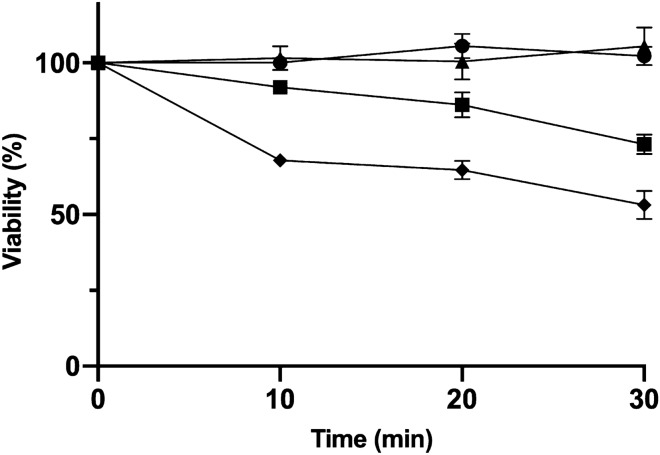
Effect of *P. maritimum* aerial part ethanol extract (PME) on *S. cerevisiae* (BY4741) viability under oxidative stress. Exponentially growing cultures were treated with 5 mM H_2_O_2_ (positive control; diamond) or 5 mM H_2_O_2_ and 250 µg/mL PME (co-treatment; square). The negative (circle) and positive controls contained the solvent of the extract and the control of extract contained 250 µg/mL PME (triangle). Colony-forming units were counted to calculate the viability (%) in each time point, assuming 100% at 0 min. Data are presented as the mean of three independent experiments ± SEM. Statistical analysis was performed between positive control and co-treatment with Student’s t-test at 10, 20 and 30 min (*p* < 0.001, *p* < 0.05 and *p* < 0.05, respectively).

Since PME revealed a strong in vitro antioxidant activity, it would be important to understand if the extract could provide an antioxidant effect in a biological context. Due to the similarities shared with cells of higher eukaryotes regarding oxidative stress response mechanisms, the yeast *S. cerevisiae* (BY4741) was selected as eukaryotic cell model to test if PME can protect cells from H_2_O_2_-induced oxidative stress. The results showed that the treatment with the solvent used in the extraction (ethanol, negative control) or with the PME, at the concentration of 250 µg/mL, up to 30 min caused no effect on cell viability (Fig. 2), suggesting absence of toxicity. Treatment with 5 mM H_2_O_2_ caused loss of cell viability over time in the positive control, whereas in the co-treatment with 250 µg/mL PME this effect was attenuated at 10, 20 and 30 min (*p* < 0.001, *p* < 0.05 and *p* < 0.05, respectively; Fig. 2). This protective effect likely results from the neutralization of H_2_O_2_, which is in line with the strong in vitro antiradical activity attributed to PME and, therefore, suggests a potential antiradical action of the extract against the radical ^•^OH formed by H_2_O_2_ and intracellular transition metal ions in the Fenton reaction. The protective effect of PME against H_2_O_2_ is in accordance with results reported by Rodrigues et al.^14^, where the co-treatment of a *P. maritimum* methanol extract with H_2_O_2_ prevented oxidative stress-induced cytotoxicity in the human neuroblastoma cell line SH-SY5Y. Moreover, a *P. maritimum* methanol extract was reported to have a higher in vitro H_2_O_2_ scavenging activity than the antioxidants α-tocopherol and butylated hydroxyanisole^13^. The capacity to scavenge H_2_O_2_ is considered a valuable feature since it can prevent the Fenton reaction that generates the highly reactive OH, which is able to damage the DNA, and overall can help control intracellular ROS levels to avert oxidative damage.

The antioxidant effects observed in our study reflect the polyphenol-rich composition of PME. Compounds related to those found in PME like catechin, epicatechin, myricetin and quercetin have been described to display higher DPPH scavenging activities than the antioxidant Trolox^38^ and to provide a high protective effect against H_2_O_2_ in cell-based assays^39–41^. Although quercetin and myricetin are present in PME in their glycosylated forms, which are usually associated with a lower antioxidant effect than their respective aglycones^42^, they are still expected to contribute significantly to the protective effect described in our study. In line with this, Rha et al.^30^ reported that a flavonol glycoside fraction from green tea induced a relevant protective effect against H_2_O_2_ in an intracellular oxidation assay in rat pheochromocytoma PC-12 cells. (Epi)catechin oligomers found in PME may also play a major role in the antioxidant effect displayed by the extract since the degree of oligomerization of (epi)catechin seems to influence this bioactivity. Accordingly, Wang et al.^28^ reported that (epi)catechin trimers revealed higher DPPH scavenging activity than monomers and dimers present in *P. paleaceum* rhizome extract and than the antioxidants ascorbic acid and gallic acid. In addition, Roig et al.^43^ reported that a procyanidin extract from grape seed, composed of monomers, dimers, trimers and higher polymers, was the most effective in decreasing the lipid peroxidation and the levels of oxidized glutathione caused by H_2_O_2_ in comparison with the respective monomers in FaO rat hepatoma cell line. Altogether, our results indicate that PME exhibits antioxidant effect in cell-free and cell-based assays, suggesting a potential to prevent oxidative stress-related damage.

### Antigenotoxicity

**Figure 3:**
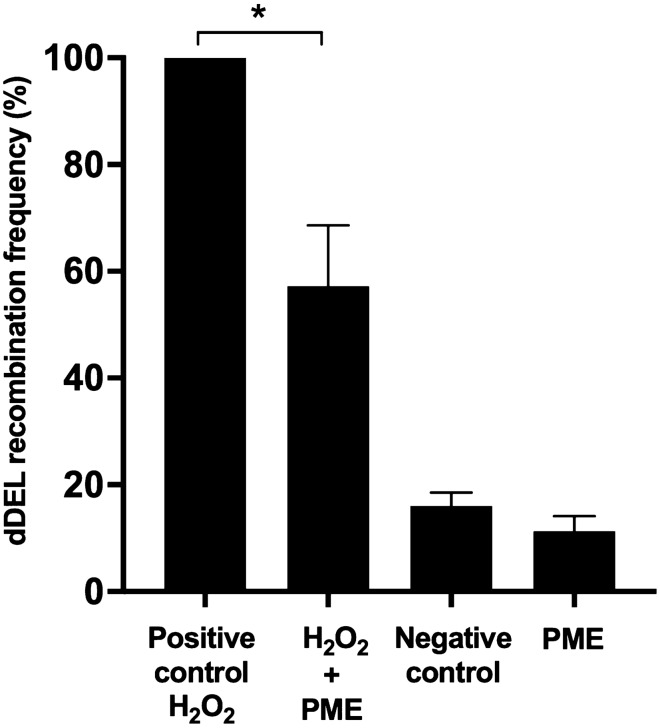
Dominant deletion (dDEL) recombination frequency after exposure to H_2_O_2_-induced oxidative stress and *P. maritimum* aerial part ethanol extract (PME) in *S. cerevisiae* (CEN.PK dDEL). Cultures were treated for 10 min with: 4 mM H_2_O_2_ in the positive control; 4 mM H_2_O_2_ and 250 µg/mL PME in the co-treatment; solvent of the extract in the negative and positive controls; 250 µg/mL PME in the control of extract. The dDEL recombination frequency (%) was calculated by dividing the number of recombinant colonies on medium supplemented with hygromycin B by the number of viable colonies on medium without hygromycin B. Data are presented as the mean of three independent experiments ± SEM normalized to the positive control. Statistical analysis was assessed between positive control and co-treatment with Student’s t-test, *p < 0.05.

Excessive levels of ROS can cause genotoxic damage, which may contribute to the first stages of carcinogenesis. Polyphenol-rich extracts with strong antioxidant properties may protect the DNA from oxidative damage and, in this way, provide an antigenotoxic effect. Thus, the potential antigenotoxicity of PME was assessed after exposure to H_2_O_2_-induced oxidative stress in *S. cerevisiae* (CEN.PK dDEL), using the dDEL assay. This assay is based on the principle that the dDEL recombination frequency increases if an agent induces DNA double-strand breaks within the dDEL cassette, which activates the homologous recombination DNA repair pathway and leads to the reversion of the *hphMX6* marker, turning the strain resistant to HygB^21^. Therefore, the dDEL recombination frequency reflects the number of DNA double-strand breaks that occurred in each treatment. The time-point 10 min was chosen in accordance with the result from the viability assay (Fig. 2) that showed the most significant protective effect against oxidative stress (*p* < 0.001). The dDEL recombination frequency observed in the negative control was considered the basal level and it was similar to that of the extract (Fig. 3), indicating that 250 µg/mL PME did not induce DNA damage. On the other hand, the treatment with 4 mM H_2_O_2_ increased the dDEL recombination frequency, implying that H_2_O_2_ caused DNA double-strand breaks (Fig. 3). This genotoxic effect was mitigated by the treatment with 250 µg/mL PME, since the dDEL recombination frequency in the co-treatment was lower than in the positive control (*p* < 0.05, Fig. 3). These results indicate that PME prevented H_2_O_2_-induced DNA double-strand breaks in a eukaryotic cell model.

Oxidative stress imposed by H_2_O_2_ led to a genotoxic effect in *S. cerevisiae*, causing DNA double-strand breaks that triggered the activation of the homologous recombination DNA repair pathway measured with the dDEL assay. The co-incubation with PME decreased the dDEL recombination frequency imposed by H_2_O_2_, suggesting a lower DNA damage and consequently the homologous recombination pathway was activated at a lower extent. This DNA protective effect provided by PME likely results from the neutralization of H_2_O_2_, preventing the Fenton reaction, or of ^•^OH before its potential interaction with the DNA. In line with this, extracts from other *Polygonum* species, *Polygonum aviculare* L. ethanol extract and *Polygonum cuspidatum* Sieb. et Zucc. ethanol and ethyl acetate root extracts, protected ΦX174 RF1 supercoiled DNA from hydroxyl radical-induced DNA strand scission (generated by UV photolysis of H_2_O_2_) in a dose-dependent manner, suggesting an in vitro DNA protective effect under oxidative stress^44,45^. Other plant extracts rich in polyphenols^46,47^ or isolated polyphenols, like quercetin^48^ and myricetin^41^, have also been reported to exhibit antigenotoxic effects against H_2_O_2_, in part, attributed to their antioxidant properties. Interestingly, a procyanidin extract revealed a stronger antigenotoxic effect against H_2_O_2_ than catechin and epicatechin, corroborating the stronger antioxidant activity suggested for oligomers^48^. Hence, PME has the capacity to prevent oxidative DNA damage.

The antioxidant and antigenotoxic properties of PME presented in our study should be further complemented with experiments performed in human cell lines and in animal models, using established methodologies to measure the redox state and DNA damage like the cell-permeable probe 2',7'-dichlorodihydrofluorescein diacetate and the single-cell gel electrophoresis assay, respectively, as these are expected to produce results closer to an in vivo situation where the extract may be consumed by humans to prevent genotoxicity.

## Conclusion

In this work, PME showed high in vitro antioxidant capacity evidenced by the low IC_50_ value determined in the DPPH radical scavenging assay, possibly linked to its high content in phenolic compounds. The antioxidant properties of PME were confirmed by the protection of cell viability against H_2_O_2_-induced oxidative stress and its antigenotoxic potential was revealed by the decrease in the dDEL recombination frequency imposed by H_2_O_2_ in the model organism *S. cerevisiae*. To our knowledge, this is the first time that a *P. maritimum* extract is reported for its capacity to protect the DNA from H_2_O_2_-induced oxidative damage, which is likely to result from the ROS scavenging activity provided by polyphenols. Our study suggests that *P. maritimum* represents a source of antioxidant and antigenotoxic metabolites with potential application in food and nutraceutical industries to mitigate oxidative and genotoxic effects such as those induced in the gastrointestinal tract by dietary xenobiotics. Thus, *P. maritimum* or its metabolites may play a role in the prevention of malignancy as a source of natural bio-antioxidants or as a functional food ingredient.

## Data Availability

All data generated or analysed during this study are included in this article.

## Abbreviations

CFU: Colony-forming unit
DAD: Diode array detection
dDEL: Dominant deletion
DPPH: 2,2-Diphenyl-1-picrylhydrazyl radical
DW: Dry weight
ESI: Electrospray ionization
GAE: Gallic acid equivalents
HPLC: High-performance liquid chromatography
HygB: Hygromycin B
IC_50_: Concentration required to inhibit 50% of the initial amount of DPPH radical
LC-DAD-ESI/MS^n^: Liquid chromatography with diode array detection and electrospray ionization tandem mass spectrometry
LOD: Limit of detection
LOQ: Limit of quantification
MS: Mass spectrometer
OD_600_: Optical density measured at 600 nm
ODC: *Ortho*-diphenol content
PME: *Polygonum maritimum* L. aerial part ethanol extract
ROS: Reactive oxygen species
TFC: Total flavonoid content
TPC: Total phenolic content
YPD: Liquid rich medium
YPDA: Solid rich medium
